# Advances and applications on non-Hermitian topological photonics

**DOI:** 10.1515/nanoph-2022-0775

**Published:** 2023-03-09

**Authors:** Qiuchen Yan, Boheng Zhao, Rong Zhou, Rui Ma, Qinghong Lyu, Saisai Chu, Xiaoyong Hu, Qihuang Gong

**Affiliations:** State Key Laboratory for Mesoscopic Physics & Department of Physics, Collaborative Innovation Center of Quantum Matter & Frontiers Science Center for Nano-Optoelectronics, Beijing Academy of Quantum Information Sciences, Peking University, Beijing 100871, P. R. China; Key Laboratory of Advanced Optoelectronic Quantum Architecture and Measurements of Ministry of Education, Beijing Key Laboratory of Nanophotonics and Ultrafine Optoelectronic Systems, School of Physics, Beijing Institute of Technology, Beijing 100081, P. R. China; Peking University Yangtze Delta Institute of Optoelectronics, Nantong, Jiangsu 226010, P. R. China; Collaborative Innovation Center of Extreme Optics, Shanxi University, Taiyuan, Shanxi 030006, P. R. China

**Keywords:** non-Hermitian topological photonics, nonlinear, quantum optics, reconfigurable, skin effect, topological phase transition

## Abstract

Non-Hermitian photonics and topological photonics, as new research fields in optics, have attracted much attention in recent years, accompanying by a great deal of new physical concepts and novel effects emerging. The two fields are gradually crossed during the development process and the non-Hermitian topological photonics was born. Non-Hermitian topological photonics not only constantly produces various novel physical effects, but also shows great potential in optical device applications. It becomes an important part of the modern physics and optics, penetrating into different research fields. On one hand, photonics system can introduce artificially-constructed gain and loss to study non-Hermitian physics. Photonics platform is an important methods and ways to verify novel physical phenomena and promote the development of non-Hermitian physics. On the other hand, the non-Hermitian topological photonics provides a new dimension for manipulating topological states. Active and dissipate materials are common in photonic systems; therefore, by using light pump and dissipation of photonic systems, it is expected to promote further development of topological photonics in device applications. In this review article, we focus on the recent advances and applications on non-Hermitian topological photonics, including the non-Hermitian topological phase transition and skin effect, as well as the applications emerging prosperously in reconfigurable, nonlinear and quantum optical systems. The possible future research directions of non-Hermitian topological photonics are also discussed at the end. Non-Hermitian topological photonics can have great potential in technological revolution and have the capacity of leading the development of both physics and technology industry.

## 1 Introduction

In quantum mechanics, the Hamiltonian with Dirac Hermitian conjugate can generally have real eigenvalues. In this case, the Hermitian conjugate can ensure that the system is closed to environment and the energy is conserved. On the contrary, if systems have complex eigenvalues, these systems are usually considered as open or non-Hermitian systems [1–4]. Until it is found in the synthetic optical systems that the refractive index of optical materials can be changed by regulating the gain and loss to achieve the non-Hermitian Hamiltonian in optics, non-Hermitian optics develops rapidly and attracts much more attention from all over the world [5–10]. Researchers focus on using quantum and artificial optical methods to study the traditional quantum mechanics and condensed matter physical phenomena from an open and broad perspective, for example, the Bloch oscillation in open systems [11]. Moreover, the non-Hermitian physics also help observe novel optical phenomena and realize a large quantity of valuable applications, which are usually based on artificial micro-nano structures such as microcavities, photonic crystals and optical waveguides [12–16]. Fortunately, one of the artificial photonic structures, designed based on topological photonics, process unique properties and provides another new degree of freedom in manipulating the photonics. Instead of regulating the frequency, wave vector, polarization, and phase of light, the properties of topological photonic systems can be changes by topological order, leading us to a novel optical world of multidimension manipulating [17–23]. These years have witnessed the flourishing of topological photonics from theory to experiments. Different optical systems within variant materials, including dielectric, metal and two-dimensional materials, have been well studied to construct topological configurations. All these models have common properties that the generated topological states are protected by the band structures and immune to the disorders. These features are conducive to retain high performance in imperfect and impure fabricated optical structures, especially for quantum systems, and there are lots of researches on them [24–29]. Due to the rich physical phenomena in topological photonics, researches gradually study open-boundary topological configurations and obtain novel optical phenomenon different from the closed-topological optical systems. These researches can be perfectly blended with non-Hermitian physics, and the non-Hermitian topological photonics has been rapidly developed [30–36]. In non-Hermitian topological photonics, the original bulk-boundary corresponding is invalid, and new theories developed by non-Hermitian situations should be used to explain the novel optical phenomena, including the topological phase transition [32, 37], [38], [39], [40], [41], [42], the skin effect [33, 43], [44], [45] and the domain wall effect [46, 47] in both open-classical systems and open-quantum systems. Moreover, based on the theoretical development, the applications on non-Hermitian topological photonics have also prospered greatly. Free space and integrated optical systems in non-Hermitian topological photonics attract more attentions, which are expected to realize high performance optical devices [48–52], excellent experimental platform [50, 53, 54] and the next-generation optical chips [55–57].

There have been many excellent review articles in the past decade to help understand the key points of non-Hermitian physics/optics and topological photonics. Non-Hermitian physical phenomena used to be realized in microcavity, which is a good platform to observe exceptional points. Cao et al. [58] and Özdemir et al. [59] have, respectively, reviewed the non-Hermitian optics, especially the configurations based on optical microcavities. Moreover, other different reviews articles focus on the basic concepts on non-Hermitian physics/optics, introducing and detailed summarizing the parity–time symmetry, exceptional points or novel phenomena such as the non-Hermitian skin effect. Feng et al. [60], Gupta et al. [56], El-Ganainy et al. [61], and Zhao et al. [62] mainly elaborated the parity–time symmetry, including the derivation process and the realized optical configurations. Miri et al. [63] and Parto et al. [64] illustrated the exceptional points in non-Hermitian physics/optics. Zhang et al. [65] has reviewed the non-Hermitian skin effect in detail. In addition, as the topological photonics developed, the field of exceptional topology/geometries of non-Hermitian physics/optics has also been well studied. Bergholtz et al. [66], Ding et al. [67] and Ghatak et al. [31] have contributed leading review articles on non-Hermitian topology. Other review articles written by Ashida et al. [68] and Wang et al. [30] are also worth reading in order to grasp key message of non-Hermitian physics and its topology. However, all these review articles do not systematically introduce the non-Hermitian topological photonics and the internal relationship with its applications. Here, we regard the non-Hermitian topological photonics as an exclusive field, accompanied by novel optical phenomena different from Hermitian case. In particular, the applications on non-Hermitian topological photonics are highlighted. Most of the examples presented in this review are emerged in the past five years, providing researchers with a convenient way to grasp the frontier progress.

In this review article, we focus on the recent advances and applications on non-Hermitian topological photonics. Section 2 summarizes the basic concept of non-Hermitian physics and topological photonics, as well as elaborates the understanding on non-Hermitian topological photonics. Section 3 illustrates the advances on non-Hermitian topological phase transition in different dimensions of optics and the realizable optical systems. Section 4 encapsulates the advances on non-Hermitian topological skin effect that has been studied in one-, two-, and synthetic dimensions. Section 5 reviews the various and valuable applications on non-Hermitian topological photonics, as well as different kinds of optical platforms including reconfigurable, nonlinear, and quantum optical systems. Finally, we summarize and outlook the challenges and opportunities on non-Hermitian topological photonics.

## 2 Non-Hermitian topological photonics

### 2.1 PT symmetry and exceptional point

Non-Hermitian physics is a broad field and many novel phenomena in non-Hermitian world, such as complex bandgap definition [69–71], topological classification [72, 73], bulk-boundary correspondence [74, 75], and skin effect [76–79], remain discussing. This section will summarize some basic concepts in order to better understand the non-Hermitian physics.

As the introduction says, a non-Hermitian Hamiltonian generally has complex eigenvalues and eigenstates, making the system difficult to study. However, the case will be much different when encountering the parity-time ( $\hat{P}\hat{T}$ ) operator. This operator is a combination of parity operator $\hat{P}$ and time reversal operator $\hat{T}$ , which operate as following

$$\hat{P}\psi_{\left(\vec{r}\right)}=\psi_{\left(-\vec{r}\right)},\;\hat{T}\psi_{\left(\vec{r}\right)}=\psi_{\left(\vec{r}\right)}^{*}$$

Notice that we are talking about boson systems so that $\hat{T}$ is just the conjugate operator $\hat{K}$ . We call a Hamiltonian $\hat{H}$ is “*PT* invariant” if it commutes with the $\hat{P}\hat{T}$ operator, i.e., $\left[\hat{H},\hat{P}\hat{T}\right]=0$ . For photonic systems where permeability $\mu_{\left(\vec{r}\right)}$ is close to one, this *PT* invariance means that the permittivity $\varepsilon_{\left(\vec{r}\right)}$ satisfies $\varepsilon_{\left(\vec{r}\right)}=\varepsilon_{\left(-\vec{r}\right)}^{*}$ . In 1998, Bender and Boettcher proposed that if a non-Hermitian Hamiltonian was *PT* invariant, its solutions may also have real eigenvalues, or the eigenvalues formed complex conjugate pairs [2]. These two cases are called *PT* symmetric and *PT* broken cases, respectively. Because $\hat{P}\hat{T}$ operator is anti-linear, $\hat{P}\hat{T}$ and $\hat{H}$ may not have common eigenstates, causing the eigenvalues non-real [57]. Generally, when the non-Hermitian part of the Hamiltonian is small, the system stays in *PT* symmetric region. In this case, the eigenvalues remain real and the eigenstates are *PT* invariant, i.e., $\hat{P}\hat{T}\psi_{\left(\vec{r}\right)}=\psi_{\left(\vec{r}\right)}$ . But if the non-Hermitian part is large compared to the coupling coefficients, the system will come into *PT* broken region. In this case, the eigenvalues become complex conjugate pairs *E*_1,2_ and the corresponding eigenstates *ψ*_1,2_ are related by the $\hat{P}\hat{T}$ operator [56, 57, 78], i.e.,

$$E_{1}=E_{2}^{*},\;\hat{P}\hat{T}\psi_{1\left(\vec{r}\right)}=\psi_{2\left(\vec{r}\right)}$$

**Figure 1:**
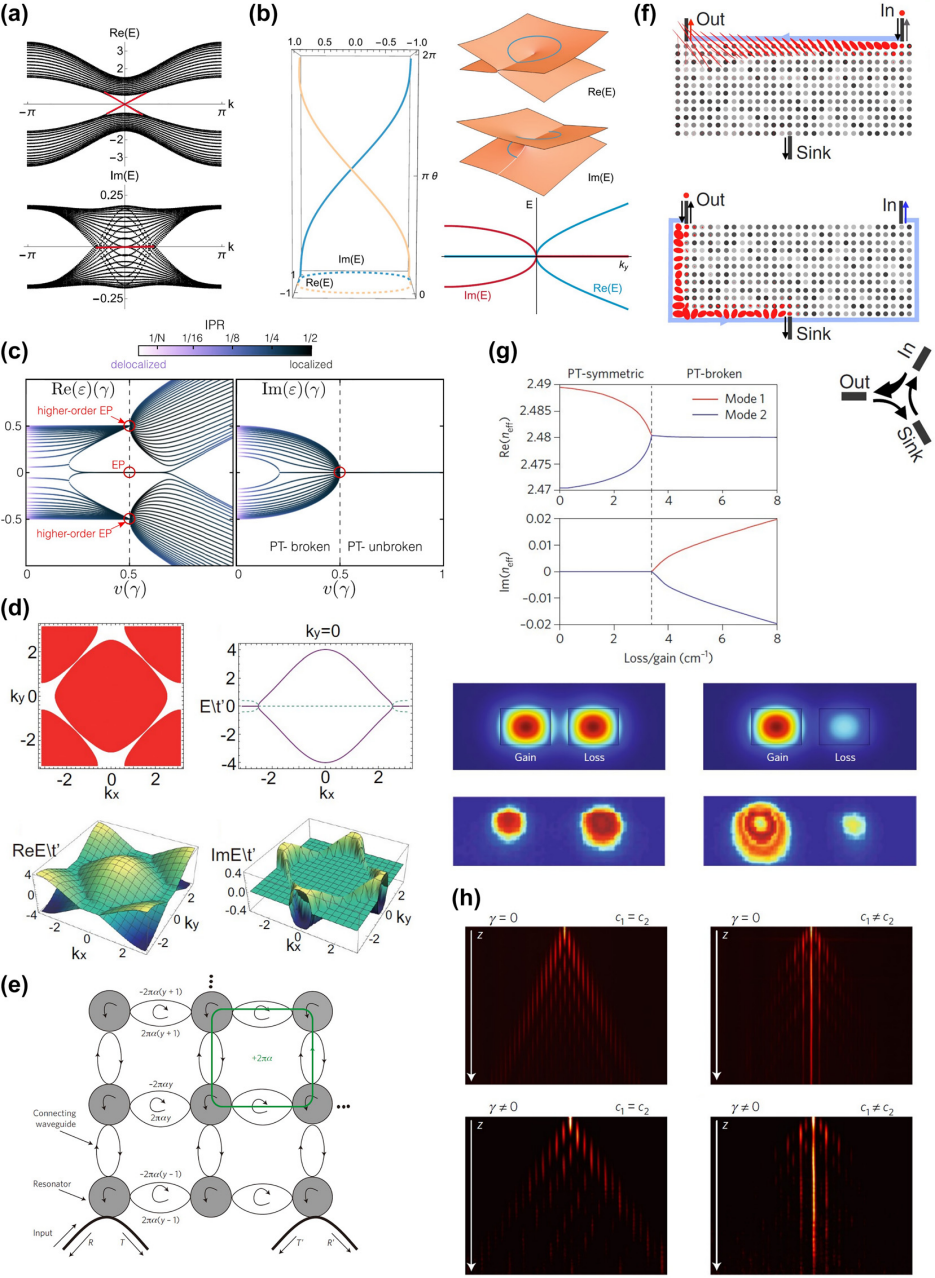
Examples of non-Hermitian physics, topological photonics and non-Hermitian topological photonics. (a) Real and imaginary part of band structures that belong to a non-Hermitian BHZ model (*N* = 32), showing purely real edge bands even in the *PT*-broken region [83]. (b) Schematic picture of the real and imaginary part of the eigenvalues closed to an EP, the left panel shows the energy evolution when cycling around the EP [69]. (c) Real and imaginary parts of the eigenvalues in a special non-Hermitian Hamiltonian (*N* = 30), showing high order EPs and anomalous localization [85]. (d) Band structure in a 2D model, the red and white regions belong to different topological numbers and are just separated by exceptional rings [86]. (e) Coupled ring resonators exhibiting synthetic gauge field and can support a pair of edge states [87]. (f) Working scene of the amplifier, only the edge states can be amplified and by wisely set source and sink one can realize nonreciprocal transport [88]. (g) *PT* invariant configuration of two coupled waveguides showing an EP, both the simulation and experimental results confirm a transition between *PT* symmetric and *PT* broken region [8]. (h) Propagation of modes in wiggled waveguide array which is equivalent to the non-Hermitian SSH model, the right bottom picture clearly shows an edge state with larger dissipation length [89].

Bender and Boettcher’s theory is of vital importance in non-Hermitian physics and has brought out a large quantity of works discussing *PT* invariant models. These models are a rather concise approach on generating real eigenvalues in non-Hermitian Hamiltonians, and can give different results from the Hermitian Hamiltonians or other non-Hermitian Hamiltonians without *PT* invariance [80–82]. For instance, by adding $i\gamma {\hat{\sigma }}_{z}$ term to the Bernevig–Hughes–Zhang (BHZ) model can lead to *PT* broken phase. The bulk states have complex eigenvalues while a pair of helical edge states have real eigenvalues, which means that only the edge states are dynamically stable [83]. The schematic is shown in Figure 1(a). Moreover, instead of adding *PT* invariant terms in primitive cell, there are also constructions of *PT* invariant boundaries, that is, adding gain to one side of the boundaries and loss to the other side. At these boundaries researchers found interface states and transition from *PT* symmetric case to *PT* broken case [84].

As mentioned above, when a non-Hermitian system is *PT* invariant, the eigenvalues can also be nonreal if the non-Hermitian part exceeds a threshold, causing a phase transition. The phase transition point, where the degeneracy happens, is known as the exceptional point (EP) [3, 66]. This special point in parameter space $\left\langle \lambda \right\rangle$ can be denoted as *λ*_0_. EPs do not only exist in *PT* invariant systems. As long as more than one energy levels degenerate and corresponding eigenstates align with each other, the point can be denoted as an EP, suggesting an incomplete eigenspace. The degeneracy of eigenvalues, namely *N*, is called the order of EP. A peculiar property is that the eigenvalue *E* changes with *λ* very rapidly close to the EP, that is, $E\propto {\left(\lambda -\lambda_{0}\right)}^{1/N}$ and 2 ≤ *M* ≤ *N* [64]. This implies that EPs are not common touching points (DPs in Hermitian physics) but actually branch points. The diagram is shown in Figure 1(b) [69]. This $\frac{1}{M}$ power law is significantly different from the linear ( $\frac{1}{M}=1$ ) or quadratic ( $\frac{1}{M}=2$ ) dispersions in Hermitian perturbation theory and can be used to detect subtle parametric changes around EPs [90]. Due to the $\frac{1}{M}$ power law, the *E*_(*λ*)_ function is multivalued and cycling an EP in parameter space $\left\langle \lambda \right\rangle$ may falls on another Riemann surface of *E*. Although the parameter *λ* and Hamiltonian *H*_(*λ*)_ both recover after a cycle, the eigenvalue *E*_(*λ*)_ and eigenstate $|\left.\phi_{\left(\lambda \right)}\right\rangle$ don’t, and they become another energy state of the same Hamiltonian, $E_{\left(\lambda \right)}^{\prime }$ and ${\left|\phi_{\left(\lambda \right)}\right\rangle }^{\prime }$ [91]. In order to recover the eigenvalue and eigenstate, one needs to keep cycling until the *E*_(*λ*)_ falls on the original Riemann surface. Once the eigenstate recovers, the topological properties such as Berry phase *γ* and winding number *w* can be defined similar to the Hermitian case.

$$\gamma_{n}=i\oint d\vec{\lambda }\cdot \left\langle \phi_{L}^{n}\right|\nabla_{\lambda }\left.\phi_{R}^{n}\right\rangle ,\;w_{n}=\frac{\gamma_{n}}{m\pi }$$

Here *n* denotes the band index and *m* is the number of cycles. The left and right eigenvectors are biorthogonal and normalized, i.e., $\left\langle \phi_{L}^{m}|\phi_{R}^{n}\right\rangle =\delta_{mn}$ . Because of the multiple cycling, the winding number is no longer restricted to integers and this is a significant difference between Hermitian and non-Hermitian Hamiltonians. Taking order-2 EP in two-band systems as an example, the energy surface consists of two Riemann surfaces (*S*_1_ and *S*_2_) and they are connected with each other [90]. This multivalued geometry originates from the square root singularity of *E*_(*λ*)_, i.e., $E_{\left(\lambda \right)}\propto {\left(\lambda -\lambda_{0}\right)}^{\frac{1}{2}}$ . If one starts from *S*_1_, after one cycle, eigenvalue *E* falls on *S*_2_, i.e., two eigenvalues exchange with each other. Only after another cycle will *E* come back to *S*_1_, causing *m* = 2. The eigenstate gains a Berry phase *γ* = *π*, corresponding to a winding number $w=\frac{1}{2}$ [85, 92]. This is the simplest non-integer winding number. If the parameter *λ* is driven by wavevector *k*, i.e., *λ* = *λ*_(*k*)_, then the *E*_(*λ*)_ relations become Bloch bands of one-dimensional (1D) system. In Hermitian physics, the winding number *w* is related to the number of topological edge states on both boundaries under open boundary condition. While in non-Hermitian physics, the $\frac{1}{2}$ winding number is related to the single edge state, implying one edge state at only one boundary [92]. The exchange of eigenvalues after one cycle also leads to the braiding of the two energy bands which belongs to the two-strand braid group *B*_2_. To describe how many times the bands enwind with each other, a topological number *ν* is defined as [35]

$$\nu =\frac{1}{2\pi i}\int_{-\pi }^{\pi }\text{d}k\partial_{k}\text{ln}\left(\det\left(H_{\left(k\right)}-\frac{\mathrm{Tr}\left(H_{\left(k\right)}\right)}{2}I\right)\right)$$

The base point of the definition is not fixed: $E_{0\left(k\right)}=\frac{\mathrm{Tr}\left(H_{\left(k\right)}\right)}{2}=\frac{E_{1\left(k\right)}+E_{2\left(k\right)}}{2}$ . It is different from the (non-integer) winding number *w* because it does not include eigenstates. The condition just mentioned above corresponds to *ν* = 1 and the two bands form a knot. If the path $\left\{\lambda_{\left(k\right)},k\in \left[0,2\pi \right)\right\}$ goes around the EP for many circles, higher *v* can be reached, which means the bands can enwind more tightly and form more knots and lines. Researchers found that the topological number *v* is related to the number of edge states at the boundary [35]. When encountering higher order EPs, namely order-*N* EPs, one may have higher root in the expression of *E*_(*λ*)_, i.e., $E_{\left(\lambda \right)}\propto {\left(\lambda -\lambda_{0}\right)}^{\frac{1}{M}}$ , 2 ≤ *M* ≤ *N*, leading to *M* cycles and $w=\frac{N-1}{M}$ [93]. The factor *M* depends on the choice of path in parameter space [94]. If the order-*N* EP is just an accidental degenerate point of several lower order EPs, then the Riemann surfaces are connected into groups. Different groups are just touched but not connected, so that the trajectory is restricted in groups and the value of factor *M* is smaller than *N*. If the order-*N* EP merges by the relation $E_{\left(\lambda \right)}\propto {\left(\lambda -\lambda_{0}\right)}^{\frac{1}{N}}$ , i.e., *M* = *N*, then all the Riemann surfaces are connected and the trajectory cycles *N* times around the EP. This is the reason that the winding number varies from $\frac{N-1}{2}$ to $\frac{N-1}{N}$ around order-*N* EP. When several EPs exist simultaneously, the Riemann surfaces become more complicated and the winding number of a specific path is determined by the EPs inside it [95–97].

In specific systems, researchers find high-order EPs whose degeneracy reaches the dimension of Hamiltonian matrix. These EPs have very large algebraic multiplicity, i.e., large energy degeneracy, but only one geometric multiplicity, i.e., one linear independent eigenvector. In this case, almost all the states are localized when the parameters are close to EPs, which is called anomalous localization as shown in Figure 1(c) [85]. Generalized EPs are exceptional rings and surfaces when encountering two-dimension (2D) and three-dimension (3D) periodic systems [98]. In these systems the Bloch wave vector $\vec{k}$ has 2 or 3 components, leading to 2D or 3D parameter space of Hamiltonian ${\hat{H}}_{\left(\vec{k}\right)}$ . These exceptional rings and surfaces can emerge from the nodal points in Hermitian systems such as Dirac points in 2D configurations and Weyl points in 3D configurations [86, 99–101]. The eigenvalues keep degenerating along these rings and surfaces, thus they form the boundary of *PT* symmetric and *PT* broken regions like EPs do. The typical exceptional rings in a 2D model are demonstrated in Figure 1(d).

### 2.2 Topological photonics

Topological physics was first discovered in condensed matter physics, starting by the experiment and interpretation of quantum Hall effect (QHE) in 1980s and opened the door of an exotic area [102–104]. It was proposed that the magnetic field could introduce nontrivial Berry curvature and led to chiral edge states, therefore, the boundaries appeared as conductor and the bulk appeared as insulator. Furthermore, it was shown by Haldane that the key role of the magnetic field in QHE was breaking time reversal symmetry [105]. Topological photonics has also developed from topological physics. In optical systems like photonic lattices, coupled ring resonators and waveguides, researchers can regulate the coupling coefficients to construct topological configurations, obtaining nontrivial bandgaps and robust topological edge states (TESs) [21, 106, 107]. One of the typical topological photonic structures is one-dimensional (1D) Su–Schrieffer–Heeger (SSH) model, usually composed of coupled waveguide or resonator arrays. When accessing to higher dimensions, topology enables unidirectional electromagnetic transport along the domain wall, which provides the possibility to guide light without back-reflections even in the existence of disorders. Besides transport properties, higher dimensional systems can also support higher-order topologies, that is, if the dimension of the whole system is *n*, then the topological states are almost confined in *n* − *m* dimensional regions (*m* ≥ 2) [108]. In 2011, Hafezi et al. proposed the pseudospin degree of freedom in optical ring resonators, i.e., the clockwise and counter-clockwise modes [87]. By tuning the phase delay in the coupling rings, they realized an artificial gauge field that resembles the field in quantum spin Hall effect. The schematic of this coupled resonator optical waveguides (CROW) arrays is shown in Figure 1(e). Based on this creative idea, researchers have studied and generalized quantum spin Hall effect in photonic systems that have much more freedom to regulate. What is more interesting is that even in the systems without specially designed periodic structures and coupling relations, there exists topological phenomena. For example, by writing the Maxwell equations in a Dirac-type form and comparing with its counterpart in quantum mechanics, researchers found that the surface Maxwell waves at the interface of two media had a topological origin. The topological invariant of this system was closely related to the sign changes of *ɛ* and *μ* across the surface [109].

As topological photonics developed, their advantages of unidirectional and robust transport promoted the TES applications in optics. Research fields on optical beam splitter, optical isolating elements, topological light emitters, amplifiers and lasers, which are very useful in light communications and integrated optical devices [21, 87], have mushroom into indispensable branches. Peano et al. used a topological photonic crystal that exhibited synthetic gauge field in order to support unidirectional topological edge states [88]. This mechanism was quite similar to the Harper–Hofstadter model in quantum Hall effect. Combining the topological configuration with parametric down conversion process, they realized the amplified edge states. This selective amplification originated from the linear dispersion of the edge state band near zero energy. They tuned the wave vector of bump light *k*_*p*_ so that *k*_*p*_/2 was just equal to the wave vector *k* of zero-energy edge state. As shown in Figure 1(f), this creative setting guaranteed the single-mode, low-noise and nonreciprocal amplification, different from that in the trivial case. Other designs based on TESs also have potential to improve the performance of optoelectronic and photonic devices, comparing to the trivial counterparts. Moreover, in addition to the applications of TESs in classical optics, the TESs can also be taken account of in quantum optics. Considering the topology with zero-point quantum fluctuations, the emission of entangled photon pairs can lead to interesting consequences. Also, some topological photonic systems are candidates for quantum computing such as non-Abelian anyon statistics [21]. Topological photonic crystals can be also combined to machine learning, for example, training the artificial neural network to get the parameters of topological photonic crystals or performing optical calculations [108, 110].

### 2.3 Non-Hermitian physics in topological photonics

Non-Hermitian physics and topology are closely related to each other, and they cause the redefinition of bandgap and reformulation of symmetries that are significantly different from the Hermitian case. Most of the topological invariants and topological phase transitions are defined only under specific symmetries. Hermitian Hamiltonians can satisfy ${\hat{H}}^{+}=\hat{H}$ , i.e., ${\hat{H}}^{*}={\hat{H}}^{T}$ , where ${\hat{H}}^{+},{\hat{H}}^{*}$ and ${\hat{H}}^{T}$ denote Hermitian conjugate, complex conjugate and transposition of $\hat{H}$ , but the non-Hermitian Hamiltonians will not. This property ramifies the symmetry classes in non-Hermitian physics. For example, the particle-hole symmetry operator $\hat{C}$ has two descendants ${\hat{C}}_{1}$ and ${\hat{C}}_{2}$ , which satisfy

$${\hat{C}}_{1}{\hat{H}}^{*}{\hat{C}}_{1}^{-1}=-\hat{H}\;and\;{\hat{C}}_{2}{\hat{H}}^{T}{\hat{C}}_{2}^{-1}=-\hat{H}$$

respectively [66]. The same thing happens to time reversal symmetry $\hat{T}$ and chiral symmetry $\hat{\Gamma }$ . At the same time, from Hermitian to non-Hermitian physics, the group of Hamiltonians is expanded and this leads to unification of symmetries. If $\hat{H}$ is Hermitian, $i\hat{H}$ is anti-Hermitian. These two Hamiltonians satisfy different symmetries but they are linear dependent, which means these two symmetries are unified in non-Hermitian physics. Researchers considered the ramification and unification of symmetries, and used similar derivations to the AZ classification in Hermitian physics [111], and they finally derived 38 symmetry classes in non-Hermitian physics [73]. These are much more than 10 original AZ classes. It should be noted that they are just fundamental symmetry classes, and there are many other symmetries not included in the 38 classes such as *PT* symmetry. Moreover, the definition of bandgap in non-Hermitian topology also needs discussion. The non-Hermitian bandgaps, i.e., the vacant regions in the complex plane, may have various geometries. There are different kinds of bandgaps and they are closely related to the topological analyzation. If a band never touches with other bands, it is called “separated” and is similar to the “gapped band” in Hermitian band theory. In this case, the topological invariants such as Chern number *C* can be well defined [69]:

$$C_{m}=\frac{1}{2\pi }\int_{BZ}i\left(\partial_{k1}\left\langle \phi_{\text{L}}^{m}\right|\partial_{k2}\left.\phi_{\text{R}}^{m}\right\rangle -\partial_{k2}\left\langle \phi_{\text{L}}^{m}\right|\partial_{k1}\left.\phi_{\text{R}}^{m}\right\rangle \right){\text{d}}^{2}k$$

*k*_1_ and *k*_2_ are two components of the wavevector. $|\left.\phi_{\text{L}}^{m}\right\rangle$ and $\left.\left|\phi_{\text{R}}^{m}\right.\right\rangle$ are left eigenvectors and right eigenvectors, i.e.,

$$\left.\left\langle \phi_{\text{L}}^{m}\right.\right|\hat{H}=\left.\left\langle \phi_{\text{L}}\right.\right|E_{m}\;and\;\hat{H}\left|\left.\phi_{\text{R}}^{m}\right\rangle \right.=E_{m}\left|\phi_{\text{R}}\right\rangle$$

Note that $\left\{|\left.\phi_{\text{L}}^{m}\right\rangle \right\}$ and $\left\{|\left.\phi_{\text{R}}^{m}\right\rangle \right\}$ are biorthogonal (i.e., $\left\langle \phi_{\text{L}}^{m}|\phi_{\text{R}}^{n}\right\rangle =\delta_{mn}$ ) [112] and both of them form complete basis as long as no degeneracy occurs. If all the bands don’t touch a baseline in the complex plane, they exhibit a “line gap”. This is a relatively strong constraint and resembles the “global gap” in Hermitian band theory. The orientation of the line (such as along the real or imaginary axis) leads to various situations protected by different symmetries [73]. If the two bands, separated by the baseline, are topological nontrivial, there will exist edge states under open boundary condition. The energy of these edge states will lie between the two bands, close to the baseline [69]. If all the bands do not touch a base point in the complex plane, they exhibit a “point gap”. In Hermitian physics, this definition has no difference from “global gap”, while in non-Hermitian physics; the energy band can wind around a point and bring the spectral winding number *w*_s_ [66]. This number describes the winding cycles of the bands around the base point, written in 1D system:

$$w_{\text{s}}=\frac{1}{2\pi i}\int_{-\pi }^{\pi }\text{d}k\partial_{k}\text{ln}\left(\det\left(H_{\left(k\right)}-E_{0}I\right)\right)$$

where *E*_0_, *I* denote the base point and identity matrix. *w*_s_ can be nonzero and indicate nontrivial topology, contributing to the topological classifications of non-Hermitian systems [73, 113]. Other elegant non-Hermitian topological physics, such as the generalized Brillouin zone and non-Hermitian skin effect which lie in the heart of non-Bloch band theory, will be further discussed in Section 4. Readers can also see these references for details [30, 31, 79, 81].

The Hamiltonian of an isolated system is always Hermitian, but for some cases, such as considering parts of the Hamiltonian that have interaction with external environment, an effective non-Hermitian Hamiltonian can be defined, which is limited to this subsystem. This conversion is usually used in open quantum systems, such as the systems with gain and loss and the systems where particles have finite lifetime [91, 114–117]. Therefore, non-Hermitian effects in optics, for instance, losses in the cavities when generating laser, are often encountered and have been considered as a negative influence in the early days. Until the non-Hermitian theory developed that people realized the huge potential of non-Hermitian photonic systems to implement novel and profitable physical processes. For example, *PT* invariant Hamiltonian can be reached by specifically introducing gain and loss in photonic devices such as optical cavities, resonators, waveguides, microwave structures, and on-chip devices, however, these optical implementations are rather difficult to realize and control in electron systems [118].

In slabby photonic lattices, there intrinsically exist non-Hermitian terms because of scattering coupling. Zhen et al. studied a 2D square lattice both theoretically and experimentally. By fitting the experimental results with theoretical formulas, they reconstructed the complex band structure near the exceptional ring [119]. Another example on intrinsic non-Hermitian optical system is strong coupling polaritons in nanoparticle chains that unavoidably suffer from radiation loss [120].

Besides considering non-Hermitian terms that already exist in optical systems, implementing gain and loss purposefully can better show the superiority and flexibility of optical systems. Tracing back to 2010, the transition from *PT* symmetric to *PT* broken region was experimentally confirmed in two coupled waveguides. In this work, non-Hermiticity was introduced by two-wave mixing in Fe doped LiNbO_3_ material. The waveguides were along *z* direction so that *z* direction mimicked the time evolution. As shown in Figure 1(g), *PT* invariant Hamiltonian was reached by diffusing Ti to make the real part of the permittivity (Re(*ɛ*)) to be symmetric along the transverse direction *x* [8], i.e.,

$$Re\left(\varepsilon_{\left(x,z\right)}\right)=Re\left(\varepsilon_{\left(-x,z\right)}\right)$$

For more complicated experimental setups, researchers added a *PT* invariant term to the SSH model and found a zero-energy interface state at the domain wall. Then they implemented it in wiggling waveguides and experimentally observed the transition from *PT* symmetric to *PT* broken cases, as well as the interface mode [89]. The transition diagrams are shown in Figure 1(h). Moreover, heading for higher dimension, exceptional surfaces with various shapes and structures can be obtained by introducing symmetric gain and loss in 3D photonic lattices [121]. In addition, the non-Hermitian terms can be added to photonic Haldane model [122], Dirac model [123], Aubry–André (AA) chains [124], Floquet temporal periodic systems [125, 126], and can be combined with topology [127–130]. Furthermore, gain and loss in photonics can also generate artificial gauge field, i.e., different amplitude between the hopping term *H*_*nm*_ and *H*_*mn*_ [52, 131]. In addition to the *PT* invariant terms, other symmetries can lead to heuristic results in non-Hermitian physics too. There are examples including inversion symmetry (*P*), particle–hole symmetry (*C*), combination of these two symmetries (*CP*) and Kramers–Kronig relation [72, 132–140]. Other novel effects such as Bloch oscillation and unidirectional reflectionless can also be investigated in non-Hermitian topological photonics [60, 63]. These research fields contain plenty of significative physics and are worthy for further studying. In next sections, the detailed advances and applications on non-Hermitian topological photonics will be illustrated, including the non-Hermitian topological phase transition, non-Hermitian skin effect and other noteworthy applications.

## 3 Non-Hermitian photonic topological phased transition

The Hermitian topological photonics has found tremendous success, while the non-Hermitian topological properties will be much richer due to the analogue of the real systems [73, 141–143]. In non-Hermitian systems, the topological properties are proven to be closely related to exceptional points and exhibit exceptional physics that has not manifested in Hermitian systems [144], which have been discussed in the previous section. The nontrivial topological features in the energy band of non-Hermitian systems provide promising pathways to achieve robust physical behaviors in both classical and quantum open systems [43]. The non-Hermitian term in photonics can be introduced by adding onsite gain and loss at different lattices to extending design of permittivity/permeability to the complex surface or by utilizing non-Hermitian hopping [144–146]. Furthermore, the induced non-Hermitian term can change the band topology in one system or even induce topological phase transition from a trivial system to a topological counterpart [147]. This section reviews the recent advances of regulating methods on non-Hermitian photonic topological phased transition.

### 3.1 Non-Hermitian topological phase transition

The topological phases of matter are one of the most important research fields in condensed matter physics. The fundamental feature is that the robust TESs are related to bulk topological invariants, which are defined in terms of Bloch band theory [34]. However, in non-Hermitian physical world with open-boundary systems, the crystals can demonstrate the non-Hermitian skin effect, and the ordinary Bloch band theory and Bloch topological invariants fail to correctly predict the energy spectra, TESs and symmetry-breaking phase transitions [148]. In order to solve the non-Hermitian topological problems, winding number and Chern number, defined by complex wave vectors, are used to characterize, for example, the non-Hermitian second-order topological insulator’s (SOTIs) topological phases in 2D (3D) photonic systems [142]. Besides, the Lyapunov exponent in the long-time behavior of bulk wave dynamics can generally reveal non-Bloch symmetry-breaking phase transitions and the existence of the non-Hermitian skin effect [148]. The complex Berry phase in non-Hermitian systems can also be used as an order parameter to characterize the associated quantum phase transition [149].

Phase transitions are generally defined as drastic changes of system’s characteristics upon a small change of a single parameter. Phase transition point connects different states of matter and the occurring of phase transition is often concomitant with the spontaneous breaking of symmetries [150]. From the view of the band structure, the band-gap closing is a signature of a topological phase transition [151]. The topological phase transition is associated with a change in the topological invariant that characterizes the band structure of the two distinct phases [152]. However, a consistent definition of topological phases in non-Hermitian was introduced, using dynamical phases instead of states of matter [72]. In this way, based on the generalized definition of topological phase, the PT phase transition in non-Hermitian system, occurring at an exceptional point, can be regarded as topological phase transition [153, 154]. In short, the phase transitions with symmetry in non-Hermitian systems describe the transition to on-average conserved energy and new topological phases [150]. New research finds that adding gain and loss to a Hermitian topological charged system allows for a new class of topological phase transition. That is, the addition of non-Hermitian perturbation transforms the Weyl points to 1D exceptional contours, while the topological charge is still preserved. Especially, when two oppositely charged exceptional contours touch, the topological charge can dissipate without opening a gap [155].

### 3.2 One-dimensional non-Hermitian models

SSH model is 1D topological configuration, which can be analyzed by tight-binding model. By alternating hopping amplitudes with gain and loss, in the non-Hermitian case, where the non-Hermitian strength *γ* is not equal to zero, the band gap gets narrow at a fixed value of *ν* which represents the tunneling amplitudes. The band gap closes and a topological phase transition occurs when the tunneling amplitudes satisfied *ω* = *ν* + *γ*. Contrary to the Hermitian case, exceptional points occur when the band gap is closed. More precisely, the two bands coalesce at the boundaries of Brillouin zone, where the eigenvalues and eigenstates become simultaneously degenerate [156]. In addition, it is found that both the hopping term and the spin–orbit coupling (SOC) can regulate the topological phase transition in the non-Hermitian SSH model [34]. The non-Hermitian term of the Hamiltonian is induced by introducing parameter *δ*, which represents the changes of different hopping strengths in the left and right directions in the unit cell.

Non-Hermitian four-band model is another 1D topological configuration with four different hopping amplitudes in each unit cell, showing different topological phases. The topological phases depend on the intensity of gain and loss with a site-dependent non-Hermitian parameter *γ*_*n*_ [151]. Through keeping the non-Hermitian parameter value unchanged and tuning the modulation strength from positive to negative values, the band structures in different gain-loss regions have different phase transition behaviors. The gain and loss distributions of the first configuration is as [+*iγ*_0_, +*iγ*_0_, −*iγ*_0_, −*iγ*_0_], where the first two sites in each unit cell have gains while the other two sites have losses. *PT* symmetry is spontaneously broken on the two middle bands, while it is retained on the top and bottom bands during the topological phase transition. For the second configuration, it is with alternating gain and loss like [+*iγ*_0_, −*iγ*_0_, +*iγ*_0_, −*iγ*_0_], and the *PT* symmetry is spontaneously broken on the top and bottom bands, the middle two bands retain *PT* symmetry during the topological phase transition. The phase transition process of these two cases is just opposite.

Aubry–Andr’e–Harper (AAH) model is known to be 1D quasicrystal topological configuration [157]. In the Hermitian case, the AAH model can be regulated as topologically nontrivial [41]. While if the AAH model with non-Hermitian *PT* symmetry, it will undergo a topological metal-insulator phase transition, known as *PT*-symmetry broken phase transition. Consider a non-Hermitian PT-symmetric extension of AAH model with complex incommensurate on-site potential on a 1D lattice. In this model, the hopping rate is represented by *T* and the amplitude of the on-site complex potential is represented by *V*_0_. Numerical results show that a metal-insulator phase transition will arise at the critical point *V*_0_ = *T*, which is signaled by a PT symmetric breaking phase transition of energy spectrum: for *V*_0_ < *T* the energy spectrum is entirely real and all eigenstates are delocalized (metallic and unbroken PT phases), while for *V*_0_ > *T* the energy spectrum becomes complex and all eigenstates are localized (insulating and PT broken phases) [158].

**Figure 2:**
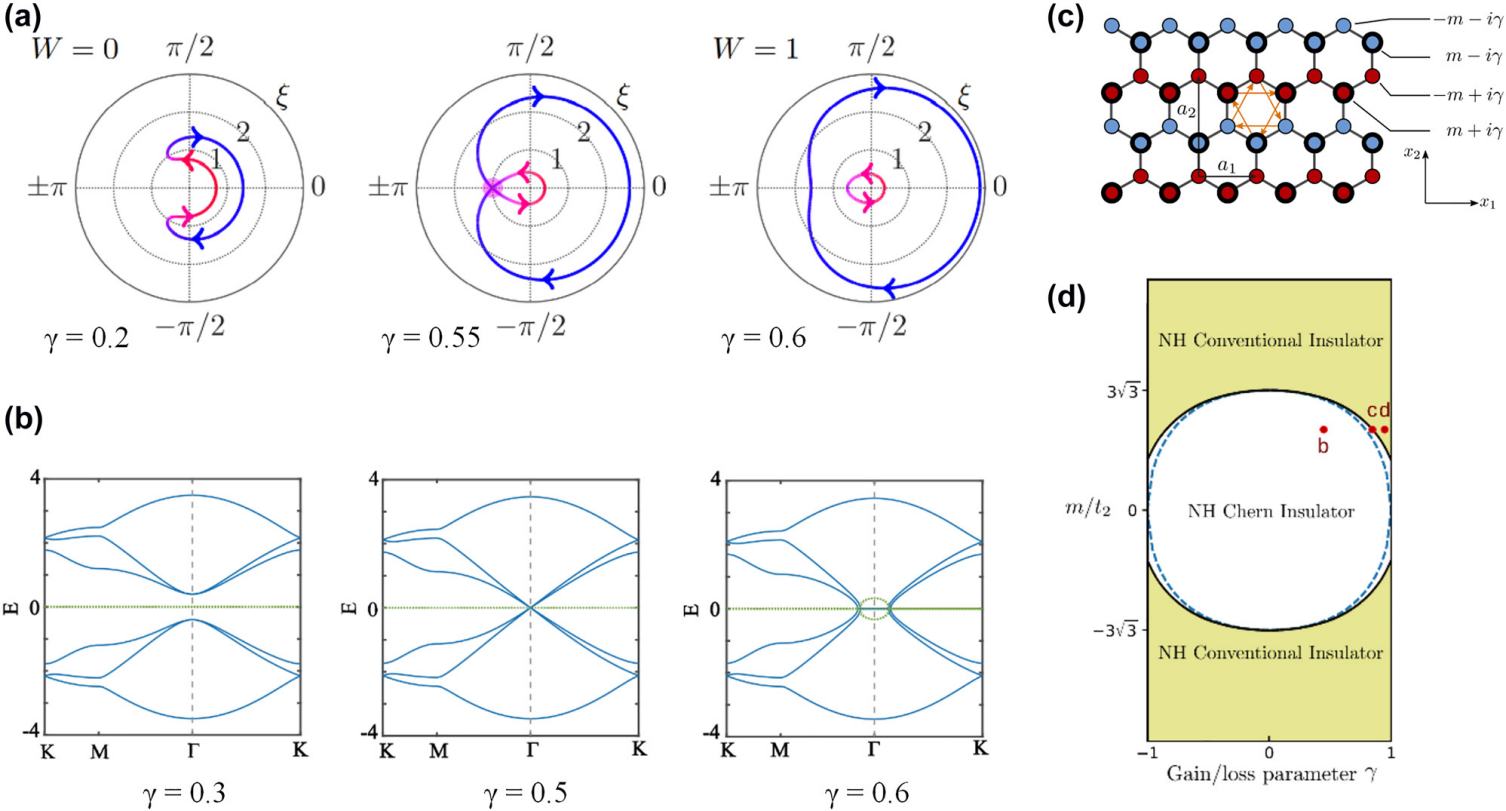
Examples of the topological phase transition. (a) Topological phase transition in the Floquet chain from the trivial phase to the nontrivial phase [159]. (b) Bulk band structures of the non-Hermitian TCI phase in the presence of the configuration of gain and loss. The spectrum becomes complex and the flat bands appear when *γ* > 0.5 [160]. (c) and (d) The 2D non-Hermitian lattices and their phase diagram, featuring a non-Hermitian Chern insulator phase (white) and a non-Hermitian conventional insulator phase (yellow) [161].

Moreover, a Floquet chain, which can split the hopping temporally, can also be considered as 1D topological model. In this model, the parameter *J* specifies the strength and the parameter *γ* specifies the directionality of hopping. In the case of *γ* > 0 (*γ* < 0), hopping to the right (left) is enhanced. By increasing the parameter *γ*, the phase of system changes from topologically trivial to topologically nontrivial [159]. The chain features a topological phase transition at the critical value of *γ*_c_ = arcosh(1/sin |*J*|). As shown in Figure 2(a), the spectrum below the transition position ( $\left|\gamma \right|<\gamma_{\text{c}}$ ) consists of a single loop, where the winding number is zero. However, at the transition point of $\left|\gamma \right|=\gamma_{\text{c}}$ , the spectrum possesses an exceptional point. Starting from the exceptional point, the spectrum splits into two loops above the transition point ( $\left|\gamma \right|>\gamma_{\text{c}}$ ), and the associated winding number is nonzero.

### 3.3 Two-dimensional non-Hermitian models

The 2D non-Hermitian topological crystalline insulator (TCI) can sit on the Kekulé texture-modulated honeycomb lattice, and this configuration can experience a phase transition by increasing the gain and loss strength *γ* [160]. As is shown in Figure 2(b), the band gap gradually decreases to zero with increasing *γ*, and bulk Dirac cones are formed at Γ point. When *γ* value is further increased, the energy spectrum develops rings of exceptional points around the Γ point, within which the real part of the spectrum exhibits flat bands pinned at zero energy, while the imaginary part has a finite value. Moreover, the Haldane-type topological phase transition, which is an exemplary phenomenon previously limited to the Hermitian regime, can be now realized in 2D non-Hermitian system. The honeycomb lattice shown in Figure 2(c). The lattice sites have complex on-site mass terms; the real parts ±*m* are indicated by thick and thin outlines, and the imaginary parts ±i*γ*, i.e., on-site gain or loss, are indicated by red and blue colors. Similarly, by increasing *γ* value from zero, the system will change from a non-Hermitian Chern insulator into a non-Hermitian conventional insulator. It should be mentioned that the system reduces to a Hermitian Chern insulator in the case of *γ* = 0. The phase diagram is shown in Figure 2(d) [161].

### 3.4 Realizable optical systems with non-Hermitian phase transition

The combination between topology and non-Hermitian in photonics holds immense potential for next-generation optical devices that are robust against defects. Most demonstrations of non-Hermitian and topological photonics have been limited to super-wavelength scales due to increased radiative losses at the deep-subwavelength scale [146]. In this section, we will show the realizable optical systems with non-Hermitian phase transitions in optical waveguide and crystal systems.

#### 3.4.1 Optical waveguide systems

**Figure 3:**
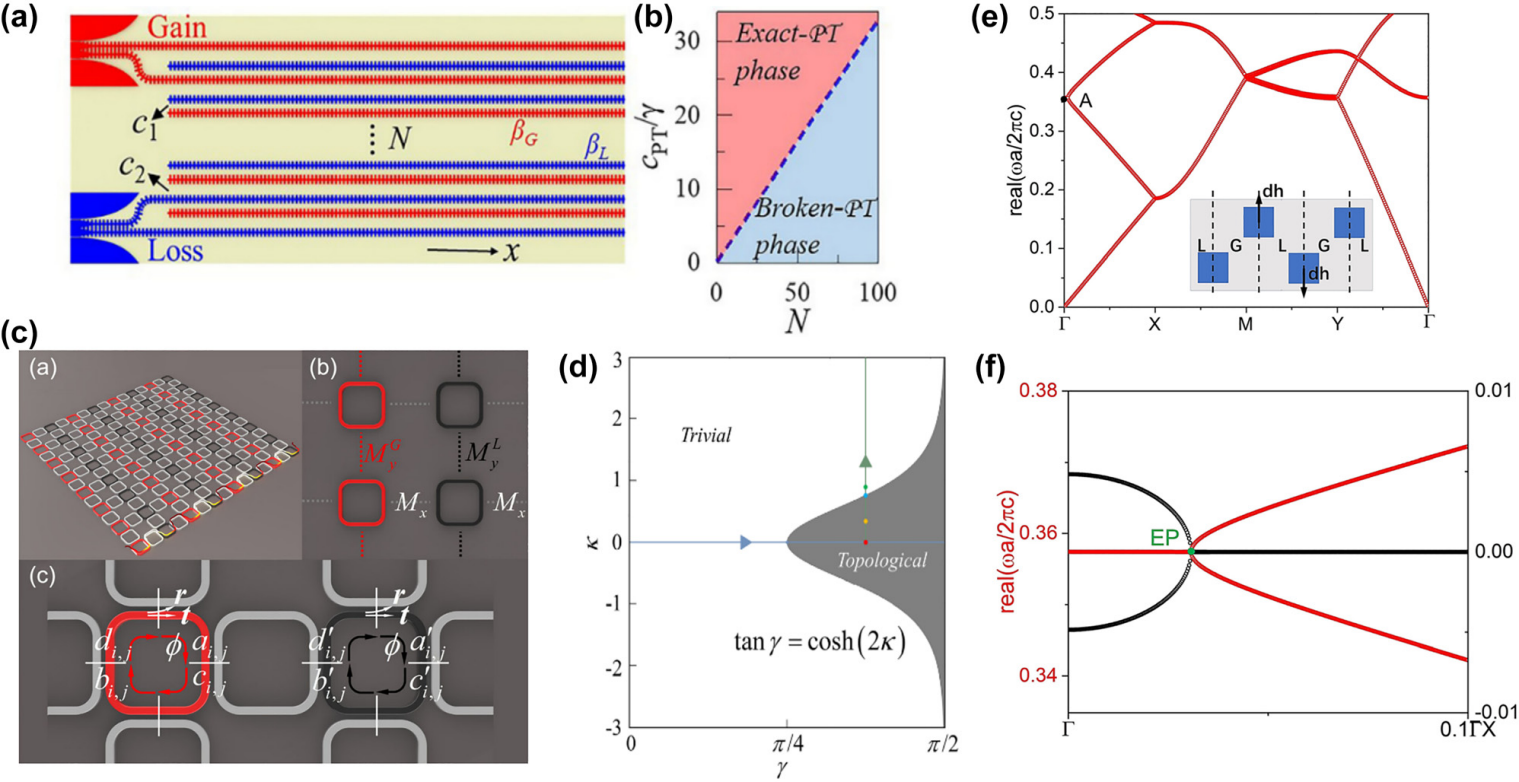
Examples of optical system to regulate non-Hermitian phase transition. (a) and (b) Model of the 1D finite non-Hermitian waveguide arrays and topological *PT* phase diagram [141]. (c) and (d) The model of non-Hermitian coupled resonator optical waveguide arrays and topological phase diagram by tuning the coupling strength and gain/loss quantity [39]. (e) and (f) The real part band structures of non-Hermitian electromagnetic double-near-zero index medium based on 2D photonics crystals, and the enlarged view of the real (red) and imaginary (black) part of the band structures near the Brillouin zone center (point A in Figure e) [163].

Coupled gain/loss optical waveguides arrayed as SSH model shown in Figure 3(a), can experience topological phase transition [141]. Consider the coupling between every straight waveguide is uniform, the topological phase of the system depends on the magnitude of the coupling coefficient *c* compared with the gain/loss strength *γ*. Increasing *c* and fixing *γ*, the system undergoes a phase transition from the *PT* broken phase to *PT* exact phase at the threshold coupling strength *c*_*PT*_, which is shown in Figure 3(b). Moreover, for an extended SSH model composed of binary waveguide arrays with alternating real and imaginary couplings, phase transition arises when the absolute values of the two couplings are equal [144]. In this case, the topological invariant of the periodic structures remained quantized with chiral symmetry even though the system was non-Hermitian. In addition, another SSH waveguide arrays, based on a Floquet modulated PT-symmetry, can also experience topological phase transition by varying the gain/loss profile and Floquet modulation frequency. The non-Hermitian modulation can induce the phase transitions between trivial and nontrivial topological Floquet states [162]. All these transitions occur at the same critical point of a single parameter. The parameter can be purely-Hermitian or purely-non-Hermitian.

The precise condition of the appearance of topological phase transition, which is decided by the coupling strength and the quantity of gain/loss in *PT*-symmetric system, has also been proved in a 2D *PT*-symmetry CROW arrays, shown in Figure 3(c) [39]. In this configuration, the topological phase transition could be realized by tuning not only the coupling strengths but also the variation of gain/loss. Ao et al. gave the analytical algebraic relation between the coupling strengths and the gain/loss strength. The topological phase transition diagram is in Figure 3(d).

#### 3.4.2 Photonic crystal systems

Triple phase transition, including topological phase transition, mobility phase transition and spontaneous PT-symmetry breaking, can be realized in a non-Hermitian Floquet quasicrystal with PT symmetry. The quasicrystal can be implemented in an optical system with controllable dissipation, consisting of coupled optical fiber loops [32]. Moreover, a unique type of a non-Hermitian electromagnetic double-near-zero index medium designed by a 2D photonic crystal occurs a phased transition in the eigenvalue spectrum. The band structure of the medium is shown in Figure 3(e) and (f). The phase transition of this system can be induced by the synergy of non-symmorphic glide symmetry of the lattice, a period-doubling of the unit cell, and the non-Hermitian perturbation of the photonic crystal [163]. Furthermore, by deliberately introducing non-Hermitian loss can induce higher-order topology in an acoustic crystal, and the bandgap can be either topological or trivial based on the configuration with losses [164].

## 4 Non-Hermitian skin effect in topological photonics

Skin effect was first found in electric conductor. When there is alternating current or alternating electromagnetic field in a conductor, the current distribution inside the conductor is uneven. The current is concentrated in the “skin” part, that is, the thin layer on the surface, of the conductor. Resulting that the current density is large when the current is close to the surface of the conductor, and the current inside the conductor is small. This phenomenon is called skin effect. While in non-Hermitian worlds, an intriguing phenomenon, known as the non-Hermitian skin effect (NHSE), has also been invested to great efforts by researchers. NHSE describes that the eigenstates of lattices with open boundaries exhibit localized behavior, which is different from the extended Bloch waves in Hermitian systems. In this section, we will explain the NHSE in details, and review the recent advances.

### 4.1 Non-Hermitian skin effect

The NHSE refers to the behavior of eigenstates of the system exponentially localized to the lattice boundary. It has profoundly changed the Bloch energy band theory, accompanying breaking the bulk-boundary correspondence in traditional topological theory, causing the differences between the open-boundary energy spectra and the periodic-boundary energy spectra. These amazing properties require a new look at the energy band theory in non-Hermitian systems, thus stimulating new researches.

**Figure 4:**
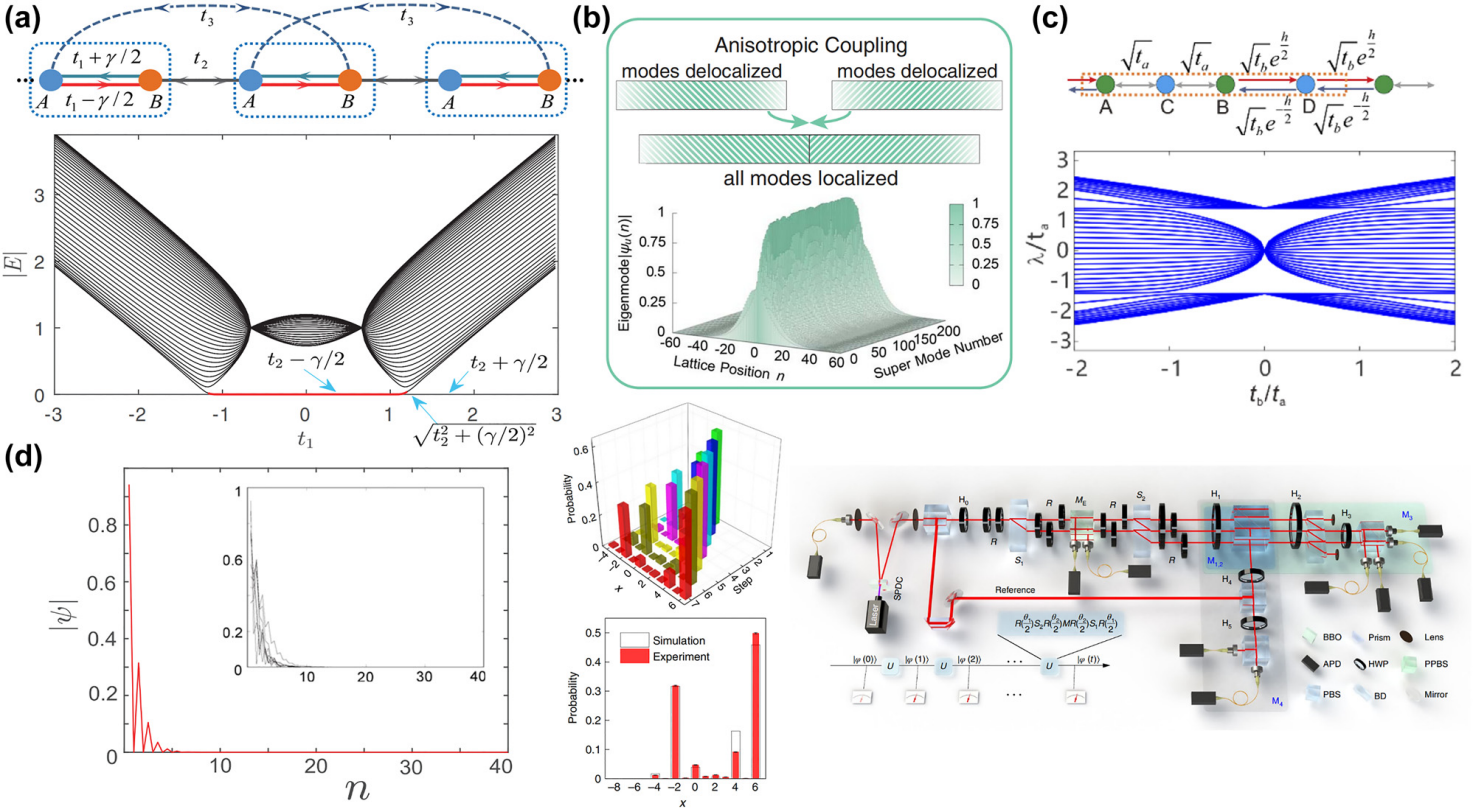
Non-Hermitian skin effect and related topological photonic configurations. (a) Non-Hermitian SSH model, non-Hermitian band structures and the eigenstate distributions of both bulk modes (inset) and edge mode. All the eigenstates are localized near the boundary [165]. (b) The anisotropic-coupling 1D model and its eigenmodes [167]. (c) The schematic and the energy spectrum for square-root SSH model [176]. (d) The experimental setup of 1D discrete-time non-unitary quantum walk and its demonstration of experimental NHSE [168].

Before further discussion, the generalized Brillouin zone (GBZ), a crucial concept in non-Hermitian physics, needs to be introduced for better understanding the NHSE. In the non-Hermitian systems, the GBZ plays the role as the Brillouin zone does in the Hermitian case, which determines some unique behaviors different from that in non-Hermitian systems. The non-Hermitian band theory based on the GBZ has also been well developed, successfully describing and predicting a large number of novel phenomena of non-Hermitian systems, which is called the non-Bloch band theory. Therefore, it is of vital importance to study the general definition and calculation method of the GBZ. The GBZ was originally proposed to explain the bulk-boundary correspondence of non-Hermitian topological states, and the redefined topological invariants in a GBZ based on NHSE can lead to largely different from the usual Bloch theory compared with the resultant phase diagrams [165]. The non-Hermitian SSH model is a typical configuration to demonstrate the topological zero modes that are determined by the non-Bloch winding number. The non-Hermitian SSH model, band structures and the diagram of the NHSE are shown in Figure 4(a). Based on the non-Bloch band theory supported by the GBZ, the NHSE can be strictly deduced and proved. Moreover, the applications of the GBZ are not only limited to characterize the topological properties, but also used to illustrate the physical properties including the non-Hermitian band structure, Green’s function, dynamics and PT symmetry [148, 166–171]. For example, the NHSE and chiral damping in open quantum systems has been studied to show that the long-time dynamics in open quantum systems can be dramatically shaped by the NHSE, which the damping is algebraic under periodic boundary conditions (PBC) but exponential under open boundary conditions (OPC) [166]. Non-Hermitian physics is such an emerging field that the researches are mushrooming in condensed matter physics [76, 172–174]. After that, a completely analytical unifying approach for studying non-Hermitian systems using generalized transfer matrices, which is suitable for both PBC and OBC, has been presented [78]. The NHSE and Anderson localization in non-reciprocal quasiperiodic lattices [175], the methods on calculating the winding number and observing the NHSE in strongly disordered non-Hermitian systems [77] have also been studied, which laid a good foundation for the theoretical improvement and applications of non-Hermite physics.

### 4.2 Advances on skin effect in non-Hermitian topological photonics

The NHSE has made great breakthroughs in condensed matter physics as mentioned above, and researchers gradually apply it to topological photonics, where the non-Hermitian terms, such as gain and loss, can be easily added. By using the gain materials or adding the scatterings to the original materials can cause abundant physical phenomena in optics [148, 177–179]. This part describes the advances on NHSE in topological photonics, as well as the optical systems with different dimensions that realize the NHSE.

#### 4.2.1 One-dimension photonic NHSE

The typical and simple topological models are usually in one dimension. They are easily calculated to analyze the physical essence, and be extended to other dimensions. Therefore, the NHSE in topological photonics is also studied in 1D topological configuration first, and demonstrates unique characteristics in both classical optical systems and quantum optical systems. For example, the NHSE in a 1D photonic lattice. The experimental setup is based on light propagation in coupled optical fiber loops, and a funneling effect for light occurred by using coupling lattices with different directions of the anisotropy. Moreover, a modified version of 1D discrete-time quantum walk is used to observe the NHSE. Due to the funneling effect relying on the NHSE and finding the huge potential of non-Hermitian world, this work then attracted huge attentions of researchers [167]. Figure 4(b) shows the anisotropic-coupling 1D model and its eigenmodes.

SSH model is used by a ring resonator array with gain and loss to investigate the NHSE in topological photonics. For instance, square-root non-Bloch topological insulators have been provided and are used to compare the differences of the band closing points between OBC and PBC, which are caused by the NHSE. These results are similar to that in condensed matter physics, but provide potential applications in optics, including light trapping, lasers, and filters [176]. The schematic and the energy spectrum for square-root SSH model is shown in Figure 4(c). Other topological configurations are also utilized in different optical conditions. The system consists of exciton–polariton elliptical micropillars, forming the 1D linear chain and circular chain, is a platform to investigate both the topological spin-Hall effect and the NHSE. The NHSE will be induced if a circularly-polarized external incoherent laser causes the imbalanced-effective decay rates of different spin polarizations, which can be applied to future robust polariton transport and the formation of multiply charged vortices [33]. In addition, the researches on topological photonic NHSE also focus on the theoretical extension. For a 1D continuous periodic model composed of photonic crystal, it can be explained by the non-Hermitian properties by using the non-Bloch band theory to realize NHSE, which corresponds well to the simulated results [180]. Despite the photonic systems, the 1D acoustic systems have been studied to demonstrate the NHSE. For example, 1D non-reciprocal acoustic crystal has been proposed to realize the NHSE and demonstrate a twisted winding that will change the NHSE, illustrating a novel modulate approach of NHSE in non-Hermitian physics [181].

In addition to the classical optical systems, the quantum optical systems are also a good platform to investigate the NHSE. As mentioned above, 1D quantum walk is a common approach to study the topological photonics by using light quantum. The observable results of quantum walk are in good agreement with the topological edge states. Consequently, the non-Hermitian optics with NHSE can also be demonstrated by quantum walk. The breaking bulk–boundary correspondence has been experimentally observed and the NHSE with bulk states by using discrete-time non-unitary quantum-walk dynamics of single photons was pronounced in 2020 [168]. Then the non-Hermitian topological Anderson insulator by using disordered photonic quantum walks was studied as well. In this case, the NHSE could also been observed, which proving the universality of the NHSE [182]. The experimental setup of 1D discrete-time non-unitary quantum walk and the experimental demonstration of the NHSE is shown Figure 4(d).

#### 4.2.2 Two-dimension photonic NHSE

**Figure 5:**
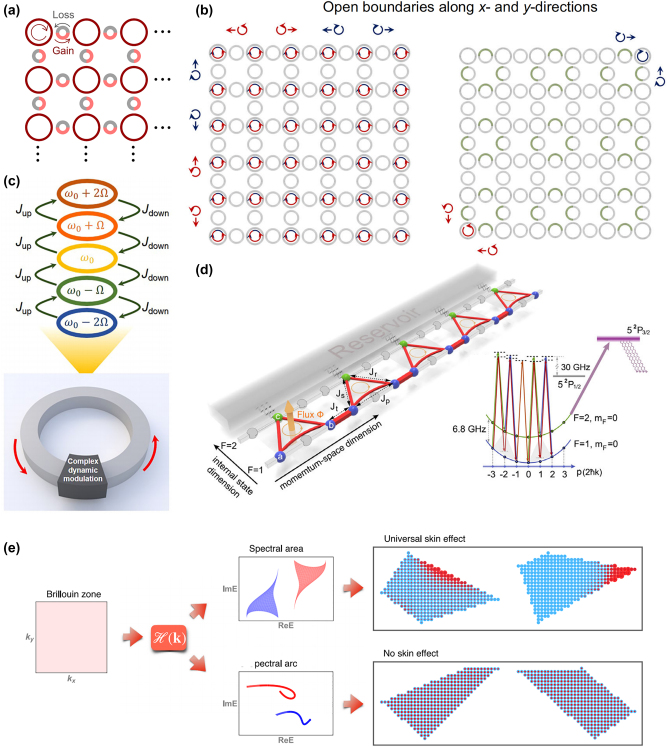
The non-Hermitian skin effect in different dimensions. (a) CROW arrays with gain and loss in 2D [185]. (b) The acoustic spin-polarized NHSE [186]. (c) Schematic of the synthetic frequency dimension with 1D NHSE [187]. (d) The topological Aharonov–Bohm chain with NHSE [188]. (e) The theorem of universal skin effect [189].

2D non-Hermitian systems usually possess more abundant physical phenomena than 1D systems. Therefore, researchers gradually turn their attention to 2D NHSE. 2D NHSE has the similar properties to 1D NHSE that the eigenmodes of bulk states will distribute like edge states, which the lattice-site intensities exponentially decay from the boundary. However, due to the flexible regulation methods in 2D systems, there can be high-order NHSE or corner modes. The nested tight-binding formalism is constructed to research the exact solutions of second-order topological zero-energy corner modes for the non-Hermitian four-band model. The corner modes induced by second-order NHSE are also been found based on this formalism [183]. Moreover, due to the extended dimension, the induced dislocations to topological configurations play an important role. It concludes that the dislocations in non-Hermitian systems can cause the density accumulation or depletion, demonstrating an essential perspective on the NHSE by separating it from the boundary conditions [184]. In addition, the CROW arrays are one of the typical topological configurations because of the analogue of quantum Hall effect. Different optical effects can be studied base on CROW. For photonic NHSE, the proposed novel method on generating single-mode laser by using nonlinear gain saturation and the NHSE is an excellent idea. The configuration is shown in Figure 5(a), the gain in pink and loss in gray were added to the coupling rings in CROW arrays. The skin mode induced by NHSE provided the opportunity for generating lasing mode [185]. Besides, the 2D acoustic higher-order topological insulators based on CROW arrays have also been reported. The central point is that the 2D NHSE lead to wave localizations toward two opposite corners for all the bulk, edge, and corner states in a spin-dependent manner in finite systems with both edges and corners. This kind of NHSE can achieve rich wave manipulation by configuring non-Hermitian properties [186]. The configuration diagram is demonstrated in Figure 5(b).

#### 4.2.3 Synthetic-dimension and higher-order photonic NHSE

Synthetic dimension is of great significance in optics developments, which can provide another regulation parameter in a photonic system. The more tunable parameters, the more functions can the system possesses. Therefore, by combining the photonic NHSE and the synthetic dimension can make not only the novel physical phenomena, but also pragmatic applications. In topological photonics, the optical frequency dimension is a common use as the synthetic dimension and then obtains frequency-dependent topological edge states. While in non-Hermitian optics, the non-Hermitian frequency lattice can also be created by introducing complex gauge potentials. By modulating the non-Hermitian lattices from spatial to frequency dimension, the obtained complex gauge potentials can cause non-Hermitian transport phenomena, as well as the non-Hermitian Bloch oscillations corresponding to the NHSE [190]. Generally speaking, a 2D photonic non-Hermitian system can be designed by adding a synthetic dimension to a 1D configuration. By tuning the spatial position and frequency mode of the light in real time of a 1D ring-resonator array, the programmable light propagation, the frequency conversion, as well as the second-order corner modes of 2D NHSE would be realized [187]. Figure 5(c) shows the 1D NHSE with synthetic frequency dimension. Moreover, the synthetic dimension can also be the ground-state hyperfine states in ultracold-atoms systems. Figure 5(d) shows the experimental realization schematic on observing NHSE in a dissipative topological Aharonov–Bohm chain based on a two-component Bose–Einstein condensate. Based on this configuration, the interplay of many-body statistics and interactions with the NHSE can be further developed [188].

In addition to the synthetic-dimension photonic NHSE, researchers also pay attention to the higher-order photonic NHSE, which bring novel physical phenomena different from lower-dimension NHSE. High-order NHSE can be considered as the eigenmodes caused by corner modes or 3D topological configurations [186]. Moreover, Zhang et al. established a theorem, explaining the 2D and higher-dimension NHSE universally, as well as the applicable conditions. The conditions in high-order dimension, different from that in 1D, are compatible with all point-group symmetries. Both the corner NHSE and the geometry-dependent NHSE were illustrated in their work [189]. The schematic of generating universal NHSE is shown in Figure 5(e).

## 5 Applications on non-Hermitian topological photonics

In the past decades, as a new field of optical science and technology, the basic theory of non-Hermitian topological photonics has been extensively and deeply developed in different systems. At present, non-Hermitian topological photonics has achieved a series of great research achievements in free space optical modulators, on-chip integrated photonic modulators, quantum system, reconfigurable system and others, and it has been already widely applied. In this section, we will focus on the major achievements and emerging applications of non-Hermitian topological photonics, and outline the challenges and opportunities of non-Hermitian topological photonics in the future.

### 5.1 Modulation of non-Hermitian topological photonics

The modulations in both free space and on-chip integrated systems are one of the main applications on non-Hermitian topological photonics. It aims to obtain high performance optical devices and search novel non-Hermitian topological phenomenon by introducing the non-Hermitian term such as gain and loss. In this section, we will review the recent progress on modulations in free space and on-chip integrated systems based on non-Hermitian topological photonics.

#### 5.1.1 Free space modulation

**Figure 6:**
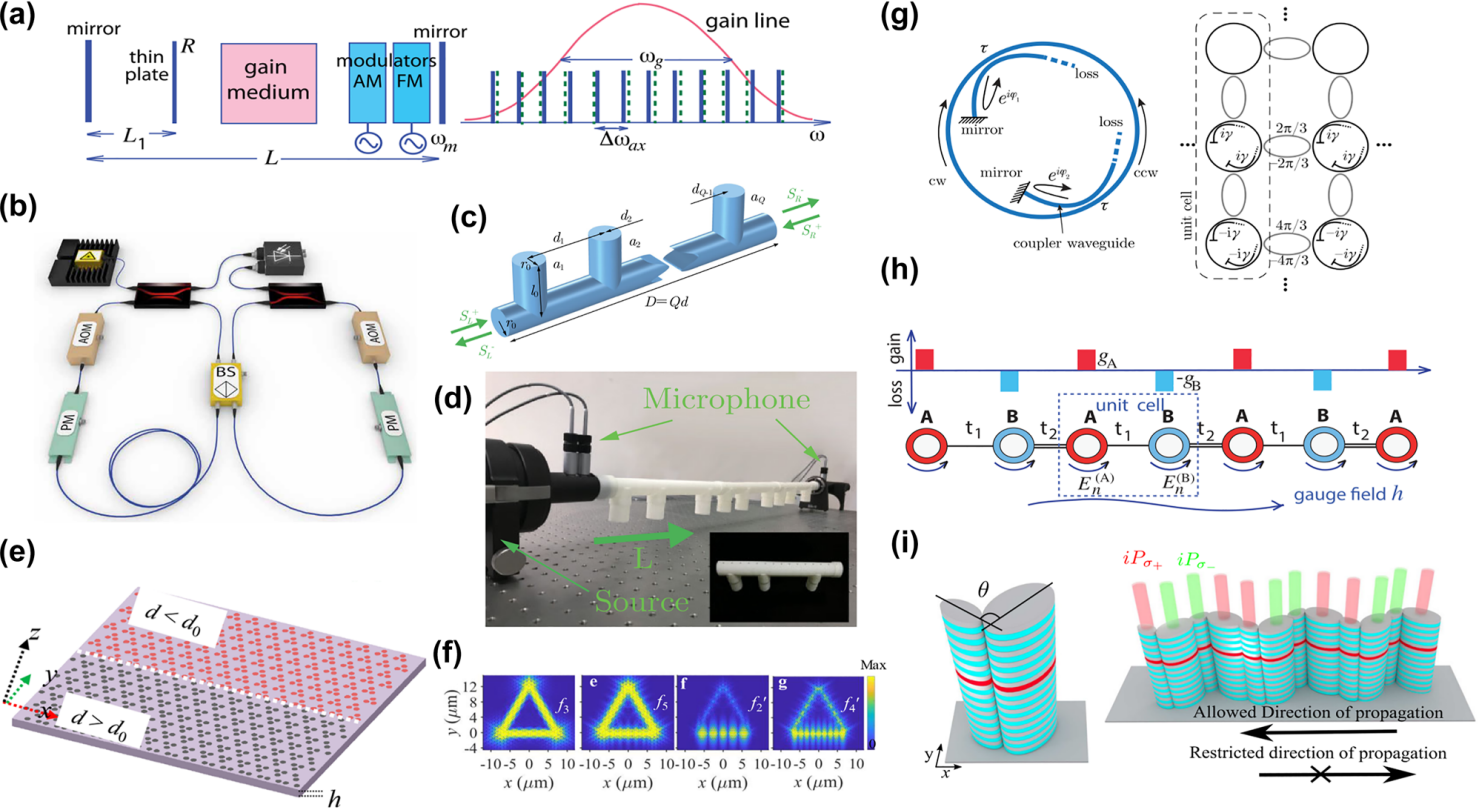
Schematics on modulation of non-Hermitian topological photonics. (a) *PT*-symmetric ML laser with incommensurate shift of cavity axial mode frequencies [40]. (b) Experimental implementation of non-Hermitian tailored photonic lattices with optical fiber loops [191]. (c) and (d) Experimental setup of a comb-like compound unit cell composed of a main tube with closed-end and side-branch tubes [192]. (e) and (f) The Kagome crystal structure and its topological mode distributions [194]. (g) Coupled ring resonator arrays and their unit cell to create a QSH system [195]. (h) Schematic of a non-Hermitian SSH lattices, which are made of coupled microring resonator arrays with alternating gain and loss [196]. (i) Elliptical micropillar pairs arranged as 1D zigzag chain [197].

The non-Hermitian modulation in free space is a common device to build a non-Hermitian physics experimental platform. As discussed before, topological phase transitions can be observed in a mode-locked laser configuration with *PT* symmetry. By introducing gain materials into the system, a non-Hermitian phase transition can be observed, as well as the actively mode-locked laser in *PT*-symmetric configuration with incommensurate spacing of cavity axial frequencies. The laser mode locking, routinely used to generate ultrashort optical pulses, could provide a fertile laboratory platform to investigate non-Hermitian phases in the spectral domain, which are shown in Figure 6(a) [40]. Free-space modulation systems can also be used to integrate optical systems. Existing research suggests that non-Hermitian photonic lattices can be realized by coupling optical fiber loops. In this system, the researchers use the coupled fiber loop to realize non-Hermitian modulation in free space and build a non-Hermitian photonic lattice, observing the shape-preserving constant-intensity waves and non-Hermitian-induced transparency, which are shown in Figure 6(b). The research also demonstrated non-Hermitian tailoring of an inhomogeneous optical mesh lattice in a photonic system [191]. Apart from photonic systems, non-Hermitical modulation in free space is applicable to acoustic systems. TESs can be constructed in a comb-like quasi-periodic acoustic structure with composite elements. If loss is introduced into the system, this non-Hermitic factor will lead to non-Hermitian physical phenomena such as an exceptional point [192]. This method, shown in Figure 6(c) and (d), provides us with a platform to study TESs in the open-boundary non-Hermitian systems, which is generally applied to wave physics [193].

#### 5.1.2 On-chip integrated modulation

With the development of micro-nano optics, the miniaturization and integration of optical devices becomes one of the main research fields for optical technologies. On-chip integrated modulation will help us achieve effective control of optical field at smaller scales and prepare more delicate optical devices. Combining the on-chip integrated photonics with non-Hermitian topological photonic can guide and help us design novel photonic devices based on the common photonic structures, including photonic crystal, optical waveguide and microcavity [51]. The non-Hermitian modulation based on photonic crystal is achieved by introducing gain and loss into the crystal structures. Some studies have shown that an anisotropic double-near-zero index medium can be constructed based on photonic crystal platform. Near the Brillouin zone center, the photonic crystals exhibit some characteristics that the real parts of its effective permittivity and permeability are simultaneously near zero, while the imaginary parts of the effective parameters are nonzero values with opposite signs [163]. Moreover, two different types of valley Hall edge patterns are realized based on the Kagome lattice, enabling the coexistence of high *Q* ring-resonator modes and lossy Fabry−Pérot resonator modes. This feature provides a powerful platform for studying non-Hermitian topological laser cavity with arbitrary geometric structures. The Kagome crystal structure and the electric field distribution are shown in Figure 6(e) and (f) [194]. In addition, it is also possible to realize non-Hermitian modulation in optical waveguide systems. The system on a chip constructs an exceptional point by utilizing the oppositely biased gyro-tropic materials with a balanced distribution of loss and gain. Besides, by operating near an exceptional point, anomalous topological wave propagation with low group velocity, inherent immunity to backscattering at discontinuities, and immunity to losses can be obtained. This proves that the system has the strong robustness in light transmission [198].

The non-Hermitic modulation can be easily designed and achieved based on the coupled ring resonators configuration. Researches have been widely carried out upon the idea. Quantum spin Hall effect can be realized in a lattice of coupled ring resonators arrays with a suitable non-Hermitian coupling between the spins. The resulting complex-eigenvalue edge state is robust against defects due to the topological protection. The schematic of the CROW arrays is shown in Figure 6(g) [195]. Furthermore, stable and phase-locked emission in an extended topological supermode of coupled laser arrays, based on concepts of non-Hermitian topological photonics, is theoretically suggested. For example, non-Hermitian SSH chains can be design by coupled microring resonators. The extended supermode, that retains the property of topological protection, can stably oscillate while suppress all other non-topological edge supermodes. The technique can be used to produce a high robust laser shown in Figure 6(h) [196]. In addition, coupled elliptical micropillars of different sizes, shown in Figure 6(i) [197], and graphene arrays [199] are also potential platforms to achieve the robust transmission, non-reciprocal transmission, sensitive sensing and the other functions based on non-Hermitian topological photonic modulations. Therefore, the non-Hermitian topological photonics provides convenience and novel ways to design and fabricate integrated optical devices, as well as the integrated optical chips.

### 5.2 Reconfigurable optical systems

**Figure 7:**
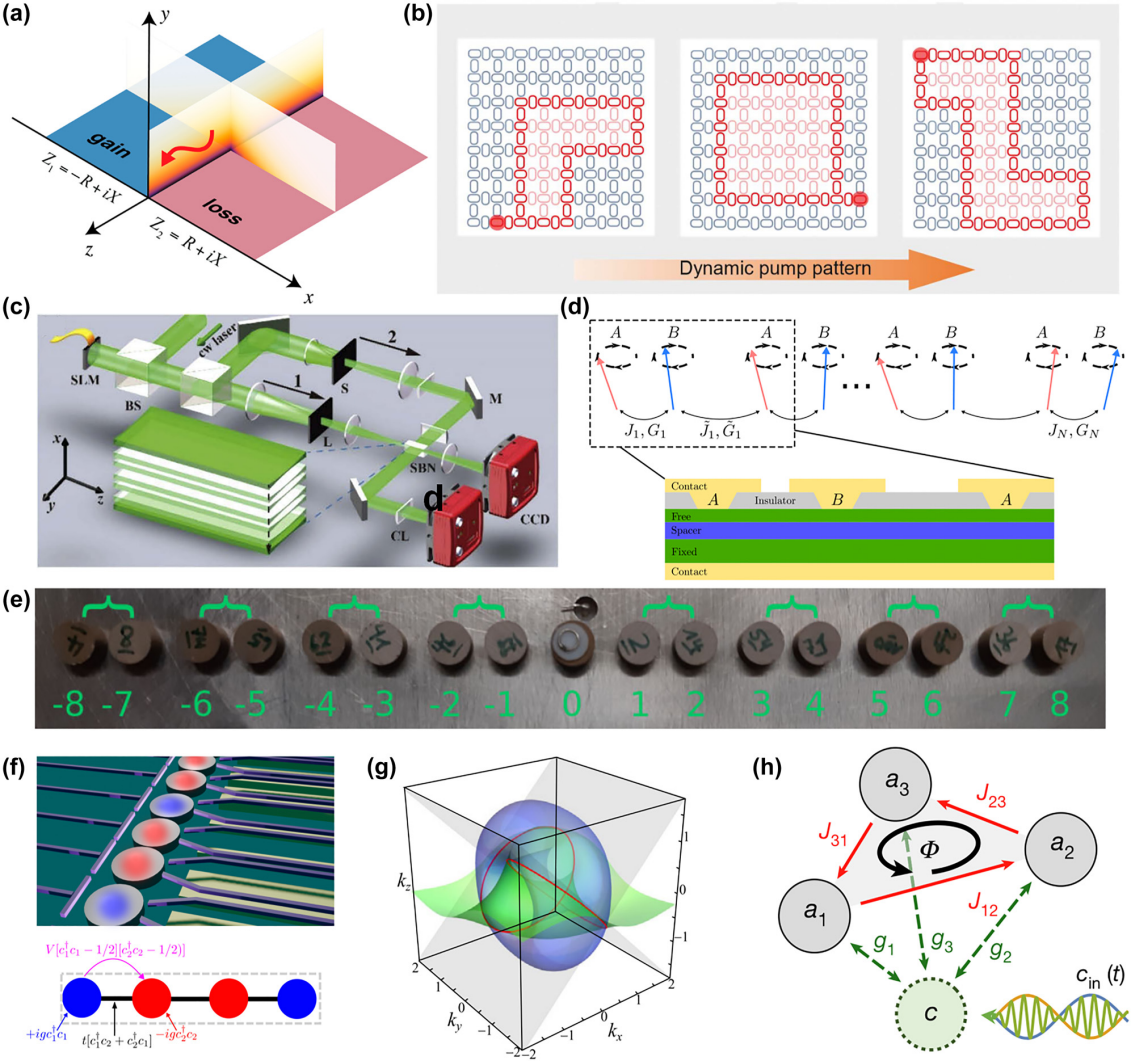
Schematics on reconfigurable, nonlinear and quantum optical systems. (a) Non-Hermitian metasurface geometry [202]. (b) Non-Hermitian control of light propagation in a topological microring lattice. Topological edge states can be dynamically reconfigured to steer light along any boundaries defined by the arbitrarily patterned pump beam [203]. (c) Experimental scheme for laser-writing photonic lattices in photorefractive nonlinear crystals [204]. (d) Schematic illustration of the array based on STOs [206]. (e) Experimental setup of SSH structure composed of dielectric resonators with an alumina plate placed on [207]. (f) Tunable non-Hermitian quantum dot chain [208]. (g) The exceptional line structure observed in experiments [209]. (h) Aharonov–Bohm interference in a Hermitian nano-optomechanical network [210].

Since light is often used as a carrier of information, the reconfigurability of optical devices will provide much more degree of freedom for regulating light. Fortunately, the non-Hermitian topological photonics is applicable to reconfigurable optical systems so that flexible and convenient regulation methods on light can be obtained. In the Hermitian system, the robust propagations of light along reconfigurable pathways are defined by synthetic gauge fields within a topological photonic meta-crystal [200], and the reconfigurable waveguides can be designed based on the topological phase transitions, tuned by thermal or electro-optic modulations, as well as the nonlinear cross phase modulation [201]. However, in non-Hermitian systems, the adjustable feature of gain and loss provides a new tunable dimension to realize a new reconfigurable system. A previous study showed that some potential applications for reconfigurable photonics and non-Hermitian topological photonics are modulated by activating the gain and loss. Even in fully passive configurations, a suitable modulation of the surface resistance may induce interesting in-plane routing responses, leading to the reconfigurable control of light field. The non-Hermitian metasurface is shown in Figure 7(a) [202]. Moreover, the reconstruction of gain and loss regions in non-Hermitian topological photonics can also construct a reconfigurable system either. In the schematic study, the pumping pattern was switched from a square shape to another shape, enabling the input beam propagating along the newly formed topological domain boundaries. This non-Hermitian controlled reconfigurable light-transportation scheme is inherently of topological robustness against defects. The light path is easily reconfigured with any shape as shown in Figure 7(b) [203]. In addition, non-Hermitian topological systems can also be achieved in the photorefractive nonlinear crystals. Because the index change in photorefractive crystals is caused by the intensity-dependent photorefractive effect, the intensity pattern of the induction beam determines what kind of index potentials can be created inside the photorefractive crystals. The lattices become reconfigurable because we can always use a white light source to “wash out” any unwanted index change and rewrite with a new intensity distribution. Existing studies have built an experimental reconfigurable platform based on photorefractive effect and 1D non-Hermitian SSH chains. The experimental scheme is shown in Figure 7(c) [204]. Furthermore, non-Hermitian topological physics can also supply a new reconfigurable photonic platform based on graphene system and photonic crystal system, which aims to realize interesting functions including imaging lattice switching with Talbot effect [205] and robust transmission of light field [48]. It has great potential for future optical field regulation through combining non-Hermitian topological physics and reconfigurable systems.

### 5.3 Nonlinear optical systems

The optical nonlinear effect exists widely, providing with a huge number of high-quality researches in optics. The applications based on nonlinear optical effects in non-Hermitian topological photonics can produce novel physical phenomena, build a new platform for the discovery of new physics and mechanisms, and provide new degrees of freedom for the design of photonic devices. 1D SSH arrays of spin-torque oscillators (STOs) offer a possible optical nonlinear configuration. STOs exhibit strong nonlinear effects, such as a nonlinear frequency shift; therefore, this platform enables the introduction of nonlinear effects into non-Hermitian 1D topological arrays. The schematic illustration of the model is shown in Figure 7(d). Based on this configuration, the influence of nonlinear effect, the effects of perturbations and exception point on topological systems can all be studied, and it has the potential to shed light onto the dynamics of a plethora of non-Hermitian systems with nonlinear effects [206]. Nonlinear gain saturated topological lasers can also be designed based on non-Hermitian topology theory. The laser therefore has the following characteristics, i.e., multimode lasing occurs at low output powers, while the pumping power beyond a certain value produces a single lasing mode, with all other candidate modes experiencing negative effective gain. This phenomenon arises in a lattice of coupled optical resonators with non-fine-tuned asymmetric couplings, and is caused by an interaction between nonlinear gain saturation and the non-Hermitian skin effect. The multi-mode property of the laser is opposite to that of the traditional laser at high pump. Due to the introduction of non-Hermitian term, the laser has the wonderful robustness. This finding might be useful for implementing high-power laser arrays [185]. Nonlinear effects can also be realized by introducing the diodes into the research system. In existing studies, the researched microwave platform consists of non-Hermitian SSH arrays with coupled dielectric resonators. A defect resonator is introduced into the middle of the chain, and it supports both linear and nonlinear losses. By coupling the central resonator with a diode through placing it above the defect resonator using a Teflon spacer, the nonlinearity can be incorporated. When the diode-induced nonlinearity is purely imaginary, the nonlinear defect mode is spectrally protected by the non-Hermitian charge-conjugation (CT) symmetry. However, the nonlinearity acquires a sizable real part for high pump powers. In this case, the defect mode is not any more protected by the CT symmetry. The experimental setup is shown in Figure 7(e) [207]. Because the most nonlinear configurations in optics and photonics are accompanied by losses, active devices are nonlinear configurations as well. As a consequence, the concepts from non-Hermitian physics are sought to provide strategies to take advantage of losses in such nonlinear materials.

### 5.4 Quantum optical systems

With the rapid development of quantum technology in the twenty first century, the science and technology about the quantum systems is rising rapidly. Some research subjects come into being the demands gradually, including finding novel quantum optical physical mechanism, building novel quantum optical research platform. The non-Hermitian topological photonics can provide us with a unique theoretical perspective, and the non-Hermitian topological mechanism in quantum system can also provide us with novel research perspectives. Therefore, studying quantum systems with the non-Hermitian topological photonics is one of the hot topics. For example, quantum dots are from the paradigmatic solid-state systems for quantum engineering, providing an outstanding tunability to explore fundamental quantum phenomena. Quantum dots, however, can be also combined with non-Hermitian topological photonics. The non-Hermitian many-body topological modes are realized in a quantum dot chain by utilizing a gate-tunable modulation of dissipation, and they emerge purely because of the non-Hermiticity. The schematic illustration of the tunable non-Hermitian quantum dot chain is shown in Figure 7(f). It is also proved that the quantum dot arrays are a great platform for engineering non-Hermitian many-body topological modes [208]. Moreover, the exceptional non-Hermitian metals with single-photon interferometry can be simulated. This is achieved by implementing nonunitary time evolution of single photons that is governed by a corresponding non-Hermitian Hamiltonian, as well as by performing interferometric measurements on the photons to extract the complex eigenenergy for each mode in reciprocal space. As shown in Figure 7(g), the experimental setup can be used to study the topological structures of non-Hermitian metallic exceptional lines [209]. Furthermore, chiral energy flow among mechanical resonators in a synthetic dimension and Aharonocv–Bohm tuning of their eigenmodes can be observed. The Aharonov–Bohm interference in a Hermitian nano-optomechanical network is shown in Figure 7(h). By introducing particle-non-conserving squeezing interactions, a non-Hermitian Aharonov–Bohm effect in ring-shaped networks in which mechanical quasiparticles experience parametric gain is obtained. This phenomenon persists down to the quantum domain, forming essential ingredients to explore new linear and nonlinear non-Hermitian topological phases. It can also point the way to exploring new non-Hermitian topological bosonic phases and applications in sensing and transport that exploit spatiotemporal symmetry breaking [210]. In addition to the above quantum optical systems, non-Hermitian topological photonics has been applied to exciton–polariton system [33] and gauge field system with an imaginary vectorial potential [211] as well. The quantum system provides a wonderful platform for the study of non-Hermitian topological photonics.

## 6 Summary and outlook

Non-Hermitian topological photonics becomes an important part of the modern physics and optics, which has penetrated into different research fields, including novel physics, quantum optics and photon technology. On one hand, photonics system can introduce artificially-constructed gain and loss to study non-Hermitian physics. Photonics platform is an important methods and ways to verify novel physical phenomena and promote the development of non-Hermitian physics. On the other hand, the non-Hermitian topological photonics provides a new dimension for manipulating topological states. Active and dissipate materials are very common in photonic systems; therefore, by using light pump and dissipation of photonic systems, it is expected to promote further development of topological photonics in applications on devices with various functions. As reviewed in this article, advances on non-Hermitian topological phase transition and non-Hermitian skin effect are promoted by the novel physics, thus stimulating the applications emerging prosperously on non-Hermitian topological photonics in different optical systems. However, this is an age that truly begins the study of non-Hermitian topological photonics. A great many of remaining problems on non-Hermitian physics, as well as the non-Hermitian optics have not been solved, such as the general bulk-boundary correspondence and the feasible approaches to realize high-performance optical devices for industrialization, which will bring a great leap to photon technology. In the next step of research, the improvement on systematic and completeness of non-Hermitian physics is a challenging but significant field, such as the research methods on calculating band structures with non-Hermitian Hamiltonian, and high-dimension topological phase transition. Moreover, searching for other possible applications based on non-Hermitian topological photonics is also a meaningful field that needs to be constantly promoted in both classical optical systems and quantum optical systems. Other free space modulations and on-chip integrated reconfigurable and nonlinear optical devices should be further considered, which are expected to undertake multi-dimension optical computing works. Non-Hermitian topological photonics has great potential in the technological revolution and has the capacity of leading the development of both physics and technology industry.
